# Perioperative complications of arteriovenous tirofiban administration versus oral dual antiplatelet therapy for stent‐assisted embolization treated aneurysmal subarachnoid hemorrhage: A retrospective, controlled cohort analysis

**DOI:** 10.1002/brb3.3439

**Published:** 2024-02-26

**Authors:** Kaishan Wang, Yujie Chen, Yao Xu, Chen Yang, Zhaopan Lai, Binbin Tan, Gang Zhu, Hongping Miao

**Affiliations:** ^1^ Department of Neurosurgery, Southwest Hospital Third Military Medical University (Army Medical University) Chongqing China; ^2^ Chongqing Clinical Research Center for Neurosurgery, Southwest Hospital Third Military Medical University (Army Medical University) Chongqing China; ^3^ Chongqing Key Laboratory of Precision Neuromedicine and Neuroregeneration, Southwest Hospital Third Military Medical University (Army Medical University) Chongqing China

**Keywords:** aneurysmal subarachnoid hemorrhage, dual antiplatelet therapy, perioperative complications, stent‐assisted embolization, tirofiban

## Abstract

**Background:**

Major perioperative complications of stent‐assisted embolization treated for aneurysmal subarachnoid hemorrhage patients include the formation of thromboembolic events (TEs) and hemorrhagic events (HEs), for which antiplatelet protocols play a key role.

**Methods:**

We conducted a single‐center retrospective analysis to compare the differences between arteriovenous tirofiban administration with traditional oral dual antiplatelet therapy (DAPT). A total of 417 consecutive patients were enrolled. General clinical characteristics, as well as the perioperative ischemic and hemorrhagic events, were retracted in digital documents. Logistic regression was conducted to identify both risk and protective factors of perioperative TEs and HEs.

**Results:**

Perioperative TEs occurred in 21 patients, with an overall perioperative TEs rate of approximately 5.04%; among these patients, the incidence of perioperative TEs in the tirofiban group was less than that in the DAPT group. Additionally, 66 patients developed perioperative HEs, with an incidence of approximately 15.83%; among these patients, the incidence of perioperative HEs was less than that in the DAPT group. No significant differences were seen between the two groups in terms of the mRS score at the time of discharge.

**Conclusion:**

This study indicated that an improved perioperative antiplatelet drug tirofiban was an independent protective factor for perioperative TEs in stent‐assisted embolization of ruptured intracranial aneurysms, but it did not impart an elevated risk of perioperative HEs and had no significant effects on the near‐term prognosis of the patients.

## Graphical abstract

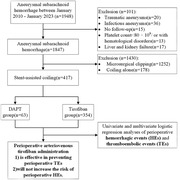

## 1 INTRODUCTION

Intracranial aneurysms represent pathologically constrained dilations of the walls of intracranial arteries, exhibiting a population prevalence of approximately 3.2%. Some studies have suggested that the ruptured intracranial aneurysm is the leading cause of subarachnoid hemorrhage, with a high lethality and disability rate, and approximately 30% of survivors are left moderately or even severely disabled (Brisman et al., 2006; van Gijn et al., 2007). Therefore, aggressive surgical treatment is the primary therapeutic modality for the rupture of intracranial aneurysms. These surgical treatments can be divided into two groups: microclamping and endovascular treatment. The 2002 International Subarachnoid Hemorrhage Aneurysm Trial (ISAT) confirmed the safety and effectiveness of endovascular embolization for intracranial aneurysms (Molyneux et al., 2002), which is currently the main surgical modality for most ruptured intracranial aneurysms, demonstrating superiority to craniotomy in some studies(Connolly et al., 2012). In stent‐assisted embolization therapy, perioperative thromboembolic events (TEs) and hemorrhagic events (HEs) are the major complications, so it is essential to explore the factors influencing their formation and actively prevent and control them. In recent years, drug resistance to traditional antiplatelet drugs, notably dual antiplatelet therapy (DAPT, aspirin and clopidogrel), has been reported in the literature, which may lead to an increased incidence of perioperative ischemic events in endovascular therapy (Gurbel et al., 2003; Wiviott & Antman, 2004). Because aspirin and clopidogrel are irreversible antiplatelet agents, the consequences of intraoperative and postoperative bleeding events can be catastrophic, and more rigor is needed when considering invasive procedures. Platelet surface glycoprotein (GP) IIb/IIIa receptor antagonists, including tirofiban, have received increasing attention because of their shorter half‐life, rapid action after administration, and faster recovery of platelet function after discontinuation. Prevention of perioperative TEs by combined arteriovenous use of tirofiban is more effective than preoperative loading dose of dual antiplatelet drugs in stent‐assisted embolization of ruptured intracranial aneurysms, and no increased incidence of perioperative HEs is observed. Therefore, in this study, we investigated the high‐risk factors for perioperative complications in stent‐assisted embolization of ruptured intracranial aneurysms and the effect of changes in the antiplatelet protocol on coagulation and platelet counts during the first day of the postoperative‐period.

## 2 METHODS

### 2.1 Study design and patients

**FIGURE 1:**
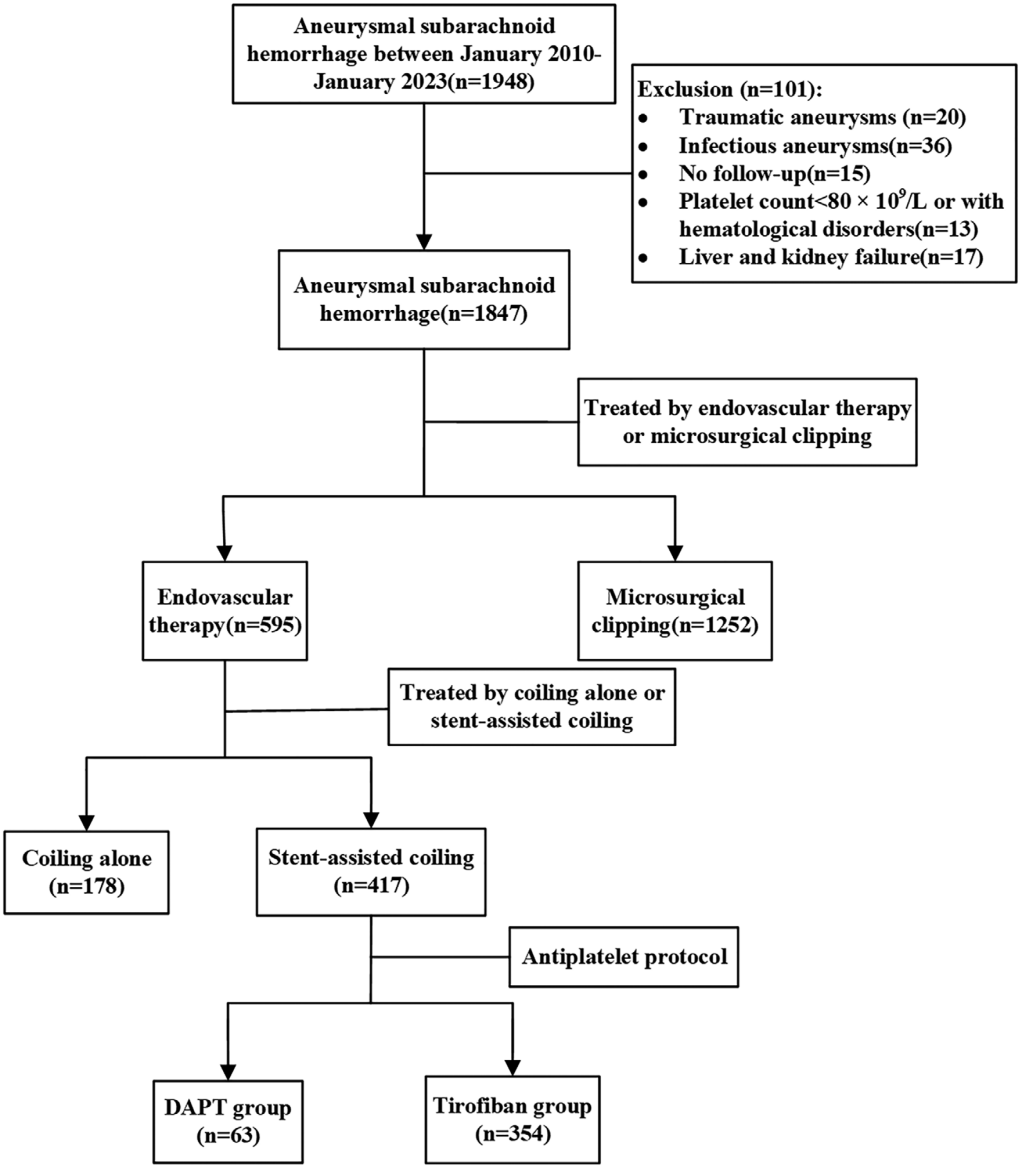
Flowchart shows the process of patient selection according to the inclusion and exclusion criteria. DAPT, dual antiplatelet therapy.

This study adhered to the Declaration of Helsinki and was reviewed and approved by the Ethics Committee of the First Affiliated Hospital of Army Medical University (No. (A)KY2022133). The demographic and imaging data of patients with subarachnoid hemorrhage in the Department of Neurosurgery of the First Affiliated Hospital of Army Medical University from January 2010 to January 2023 were retrospectively collected. The patients were divided into a loaded oral DAPT group (*n* = 63) and an arteriovenous tirofiban administration group (*n* = 354) according to the perioperative antiplatelet protocols used by the patients. Data from 417 patients were collected (Figure 1).

### 2.2 Procedures

Cerebral angiography was performed for all patients under general anesthesia, and 3D reconstruction was used to determine the working angle and to measure the aneurysm size and diameter of the aneurysm‐carrying artery. The procedure was performed under roadway fluoroscopy, and each patient was given 50–70 IU/kg of heparin intravenously at the beginning of the procedure to maintain an intraoperative activated clotting time (APTT) of 250–300 s. An additional 1000 IU of heparin was given every hour to maintain the activated clotting time. The procedure was performed by one of three different operators who was chosen randomly. All ruptured aneurysms were subject to stent‐assisted embolization if they were wide‐neck aneurysms. The stents and coils were selected by one of the three operators at random, and stent‐assisted coil embolization was performed with a semi‐release technique during the procedure. After the procedure, the grade of embolization was evaluated by multi‐angle angiography, and 3D imaging. Dyna CT was performed immediately after embolization to determine the occurrence of intraoperative bleeding.

### 2.3 Antiplatelet protocols

The 417 recruited patients were divided into a loading DAPT group and a tirofiban group according to the perioperative antiplatelet protocol used for the patients. 63 patients admitted from January 2010 to January 2016 were given 300 mg of aspirin and 300 mg of clopidogrel orally preoperatively (patients who could not take the drugs orally were given the drugs via gastrostomy tube), and 354 patients admitted from 2016 to January 2023 were given tirofiban without the need to take dual‐antiplatelet therapy orally preoperatively. Intraoperatively, 10 mL of tirofiban was pushed via a guiding catheter after stent semi‐release and pumped intravenously at 0.1 μg/kg/min and maintained at 0.1 μg/kg/min for 24 h after the end of the procedure, with a bridging oral dose of 100 mg of aspirin and 75 mg of clopidogrel 6 h prior to the end of the tirofiban pumping. All patients received regular daily oral doses of 75 mg clopidogrel for 3 months and 100 mg aspirin daily for 6 months after surgery.

### 2.4 Definition of perioperative TEs and HEs

Perioperative TEs were defined as any angiographic filling defect of the aneurysm‐carrying artery or distal arteries during DSA examination and treatment or new ischemic symptoms such as consciousness changes and limb movement changes in the 72‐h postoperative period with new low‐density shadows or ischemic spots in the vascular distribution area (excluding vasospasm) as clarified by CT or MRI (Wang et al., 2020). Perioperative HEs were defined as contrast extravasation or herniation of the coil out of the aneurysm capsule during DSA examination and treatment; new intracranial hemorrhage with or without certain clinical symptoms on postoperative head CT/MRI; and other severe bleeding events, such as severe gastrointestinal bleeding, respiratory bleeding, and multiple skin petechiae ecchymoses (Wu et al., 2021).

### 2.5 Data collection

Patient demographic and clinical information, such as age, sex, preoperative venous blood platelet count, aneurysm location, previous medical history, and history of smoking and alcohol consumption, was retrospectively collected. Coagulation counts and venous blood platelet counts were determined within 24 h after surgery. A bedside CT scan was performed immediately after the operation to detect any newly issued blood conditions during the operation, and a routine CT scan was performed 1 week after the operation to check the absorption of subarachnoid hemorrhage and the presence of asymptomatic ischemic events. If the patient developed limb weakness or other neurologic symptoms, CT scanning was performed immediately. After the exclusion of newly issued hemorrhage, CT perfusion imaging could be performed according to the severity of neurological symptoms, and MRI could be performed to clarify the presence or absence of ischemic foci if significant areas of hypoperfusion were detected. The modified Rankin Scale (mRS) score was calculated for all patients at discharge, and an mRS score of ≤2 was considered a good clinical outcome.

### 2.6 Statistical analysis

Statistical analyses were performed by using SPSS 26.0. Continuous variables that conform to a normal distribution are expressed as the mean ± standard deviation, while qualitative and hierarchical variables are expressed as values and percentages. Categorical variables were analyzed using the chi‐square test, continuity correction, and Fisher's precision test. Continuous variables were tested using the *t*‐test and the Wilcoxon rank‐sum test. We compared the baseline characteristics, including age, sex, preoperative platelet count, aneurysm location and size, number of aneurysms in a single treatment, and previous medical history, between the two groups. Missing data accounted for <5% of all data; therefore, only variables with complete data were analyzed in this study. Univariate and multivariate logistic regression analyses were performed to identify factors associated with perioperative TEs and HEs. Factors with a *p*‐value <.1 in the univariate analysis were entered into the multivariate regression analysis. Odds ratios (ORs) and 95% confidence intervals (CIs) were calculated. A two‐sided *p*‐value < .05 was considered to indicate statistical significance.

### 2.7 Clinical trial registration

The present study was registered in the Chinese Clinical Trial Registry (No. ChiCTR2200067188).

## 3 RESULTS

In this study, a total of 417 patients were enrolled, 124 male (29.5%) and 293 (70.5%) female, and 478 aneurysms were treated. The baseline characteristics of all the patients and aneurysms are shown in Table 1. There were no statistically significant differences between the two groups in terms of the baseline characteristics.

**TABLE 1:** Baseline characteristics of enrolled patients.

Variables	DAPT (*n* = 63)	Tirofiban (*n* = 354)	*p*‐Value
Sex (Female/Male)	42/21	251/103	.498
Age > 75	9 (14.3%)	28 (7.9%)	.101
Current smoking	13 (20.6%)	75 (21.2%)	.921
Current drinking	12 (19.0%)	88 (24.9%)	.320
Hypertension	33 (52.4%)	202 (57.1%)	.490
Diabetes	8 (12.7%)	46 (13.0%)	.949
Atherosclerosis	29 (46.0%)	166 (46.9%)	.900
WFNS grade			.541
Grade I–II	5	37	
Grade III–V	58	317	
Preoperative WBC	10.16 ± 4.02	10.56 ± 4.00	.493
Aneurysm size (mm, ± SD)	5.47 ± 3.36	5.02 ± 2.20	.911
Preoperative platelet count	205 (153–252)	197.5 (156–245)	.842
Aneurysm location			.953
Anterior circulation	55 (87.3%)	310 (87.6%)	
Posterior circulation	8 (12.7%)	44 (12.4%)	
Dome‐to‐neck ratio < 2	60 (95.2%)	336 (94.9%)	1.000
Procedure time>4.1 h	7 (11.1%)	42 (11.9%)	.864
Number of treated aneurysms (1/2/3)	53/7/3	312/36/6	.290

Abbreviations: DAPT, dual antiplatelet therapy; WFNS, World Federation of Neurological Society.

Among the 417 patients, 21 patients experienced perioperative TEs, resulting in an incidence of approximately 5.04%. Within the DAPT group, seven patients experienced intraoperative thrombosis or symptomatic cerebral infarction after surgery, resulting in an incidence of approximately 11.11% for perioperative TEs. Within the tirofiban group, 14 patients experienced intraoperative acute thrombosis or symptomatic cerebral infarction after surgery, resulting in a perioperative TE incidence of approximately 3.95%. A significant difference was observed between the two groups (*p* = .037 < .05), as shown in Table 2. In total, 37 patients experienced perioperative HEs, with an incidence of approximately 8.87%. In the DAPT group, 10 patients (15.87%) developed perioperative HEs. Perioperative HEs were observed in 27 patients (7.63%) in the tirofiban group, among whom 15 had intraoperative aneurysm rupture‐related hemorrhage and 22 had other hemorrhages, including multiple petechial ecchymoses of the skin, pulmonary hemorrhage, persistent hematuria, and new gastrointestinal bleeding; there was a difference in the incidence of perioperative HEs between the two groups (*p* = .034 < .05), as shown in Table 3. There were significant differences in the activated partial thromboplastin time (APTT), prothrombin time (PT), and thrombin time (TT) within 24 h after surgery between the two groups of patients treated with stent‐assisted embolization (*p* < .05), as presented in Table 3. However, no significant differences in fibrinogen (Fib) and venous blood platelet counts were observed between the two groups within 24 h after surgery (*p* > .05), as shown in Table 4. It was found that the use of tirofiban resulted in less postoperative coagulation than DAPT. The results were consistent with both groups of perioperative high‐risk patients who showed a reduced risk of hemorrhage.

**TABLE 2:** Perioperative thromboembolic event (TE) complications.

Groups	TEs		Total
	Yes	NO	
DAPT	7 (11.11%)	56 (88.89%)	63
Tirofiban	14 (3.95%)	340 (96.05%)	354
Total	21 (5.04%)	396 (94.96%)	417

Abbreviation: DAPT, dual antiplatelet therapy.

**TABLE 3:** Perioperative hemorrhagic event (HE) complications.

Groups	HEs		Total
	Yes	NO	
DAPT	10 (15.87%)	53 (84.13%)	63
Tirofiban	27 (7.63%)	327 (92.37%)	354
Total	37 (8.87%)	380 (91.13%)	417

Abbreviation: DAPT, dual antiplatelet therapy.

**TABLE 4:** Postoperative coagulation indicators and venous platelet count.

Variables	DAPT (*n* = 63)	Tirofiban (*n* = 354)	*p*‐Value
APTT	31.6 (22.6–47.52)	25.8 (24–28.3)	.001
PT	12.1 (11.57–13.00)	10.9 (10.4–11.6)	.000
Fib	2.93 (2.225–3.56)	2.73 (2.15–3.48)	.561
TT	20.1 (15.6–26.25)	16.3 (17.6–19.4)	.007
Platelet count	183 (149–226)	181.5S (149–222)	.787

Abbreviations: APTT, activated partial thromboplastin time; PT, prothrombin time; TT, thrombin time; Fib, fibrinogen; DAPT, dual antiplatelet therapy.

**FIGURE 2:**
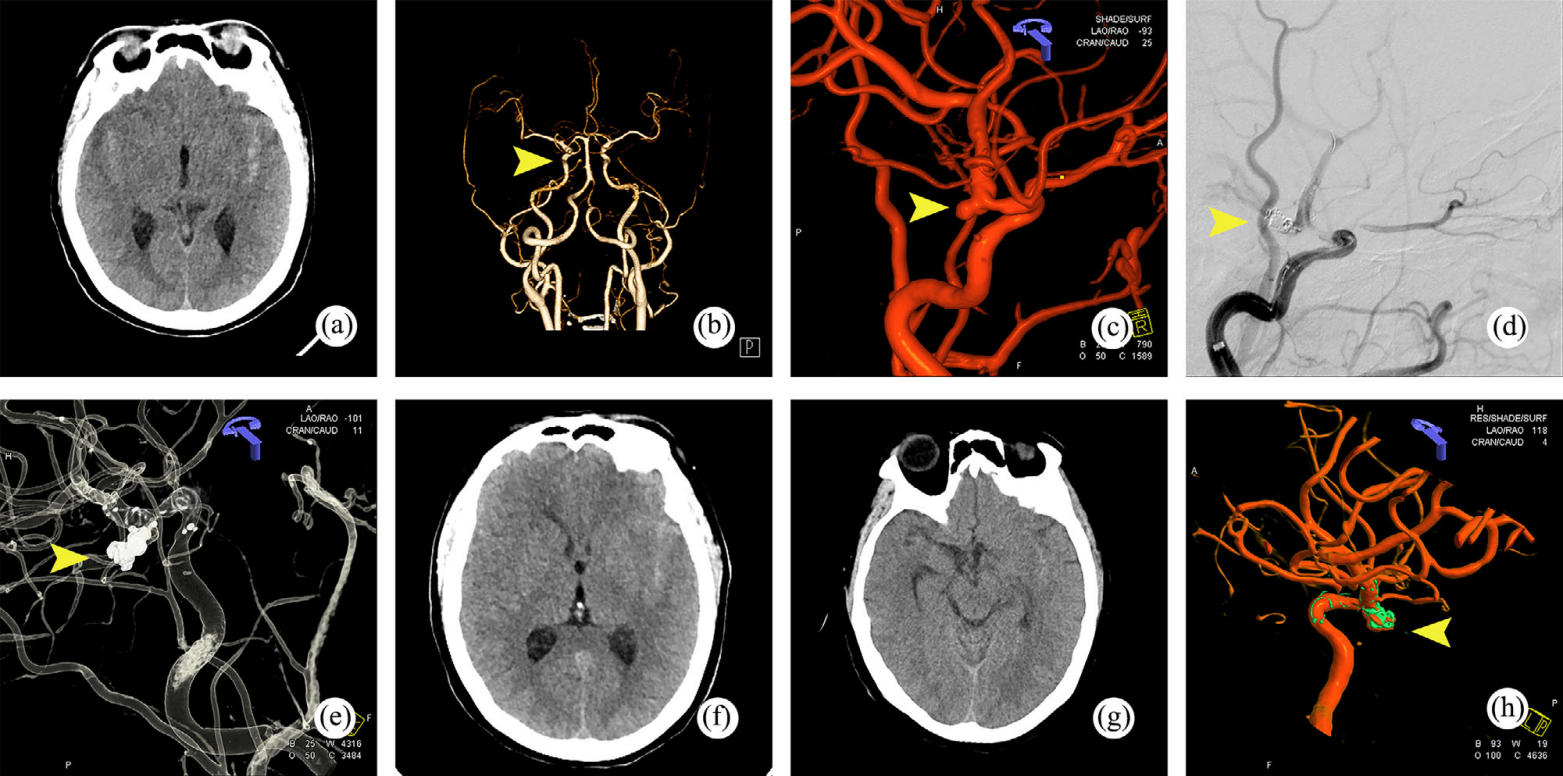
Representative case of subarachnoid hemorrhage due to a ruptured aneurysm on the C7 segment of left ICA. (a,b) Subarachnoid hemorrhage due to a ruptured aneurysm on the C7 segment of the left ICA on brain CTA. (c) Left ICA aneurysm identified by cerebral angiography with 3D reconstruction. (d) During embolization, contrast medium leakage was observed, possibly due to bleeding following aneurysm rupture. (e) Continued embolization leading to dense embolism. (f) Brain CT scan on the day of the surgery did not reveal any additional bleeding. (g) The bleeding was almost completely absorbed 8 days after the operation. (h) No evidence of in‐stent stenosis was observed during the cerebral angiography 3D imaging review conducted 8 months after surgery. ICA, internal carotid artery; CTA, computed tomography angiography.

**FIGURE 3:**
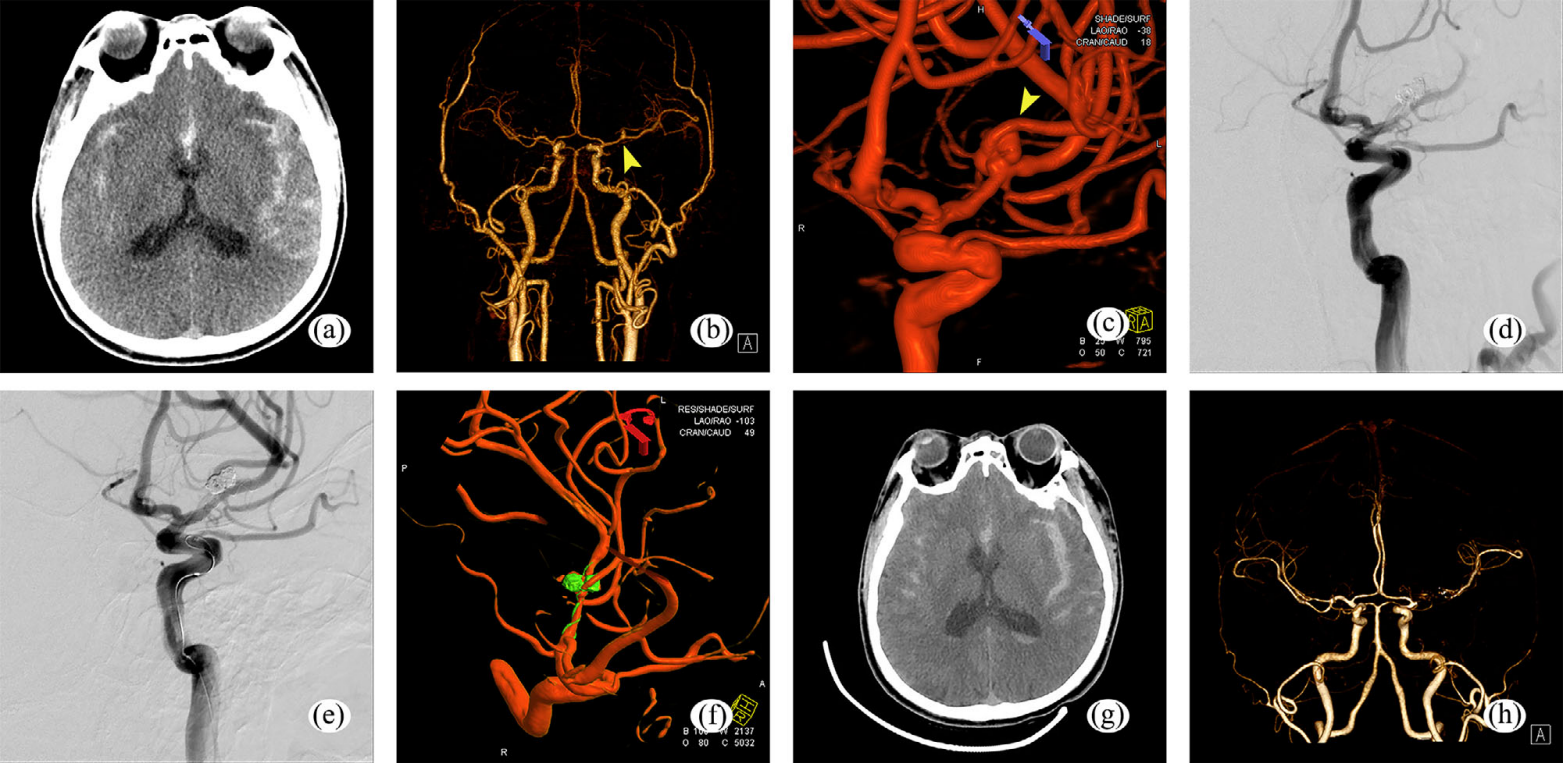
Subarachnoid hemorrhage due to a ruptured aneurysm on segment M1 of the left MCA. (a,b) Brain CTA scan indicating a subarachnoid hemorrhage resulting from a ruptured aneurysm on segment M1 of the left MCA. (c) Cerebral angiography performed to clarify the aneurysm in the M1 segment of the left MCA. (d) Acute thrombosis of the M2 branch of the left MCA was considered when it could not be visualized during the stent‐assisted embolization. (e) Visualization of MCA branches after repeated tirofiban injections and intravenous pumping. (f) MCA branches are clearly visualized on cerebral angiography with 3D imaging. (g,h) Brain CTA performed on the sixth day after surgery showed a distinct bifurcation of the left MCA and clear branching vessels distal to the bifurcation.MCA, middle cerebral artery.

The mRS scores at discharge of patients who developed perioperative ischemic and hemorrhagic complications were 0–2 in 34 cases, 3–5 in 17 cases, and 6 in 4 cases. Among patients who developed complications during the perioperative period, those in the two groups showed no significant difference in the mRS score (*p *= .642 > .05), as shown in Table 5. In the tirofiban group, a 65‐year‐old female patient was admitted to the hospital with a subarachnoid hemorrhage caused by the rupture of an aneurysm located in the C7 segment of the left ICA (Figure 2). During the embolization procedure, extravasation of contrast media was observed, suggesting an aneurysm rupture. A swift occlusion of the aneurysm was performed, and there was no further aneurysm bleeding after the completion of the occlusion. No increase in hemorrhage was observed on repeat postoperative CT scans on days 1 and 8 postoperatively (Figure 2). The patient was successfully discharged from the hospital 22 days after the operation with an mRS score of 0 at the time of discharge. In a 65‐year‐old man with a ruptured aneurysm of the left middle cerebral artery M1 segment that caused subarachnoid hemorrhage (Figure 3), the left middle cerebral artery M2 branch was not clear, likely due to acute thrombosis during surgery. The patient received several arterial injections of 20 mL tirofiban through the catheter during the operation. Subsequent imaging indicated clear M2 segment branches of the middle cerebral artery and successful recanalization. Follow‐up CTA scans did not indicate any noticeable cerebral infarction, and the distal vessels of the middle cerebral artery were displayed (Figure 3). The patient was successfully discharged from the hospital 22 days after the operation, with an mRS score of 0. Comparing the mRS scores of all patients at the time of discharge (Table 6). There was no significant difference between the two groups (*p* = .637 > .05).

**TABLE 5:** Modified Rankin Scale (mRS) score of patients with perioperative complications at the time of discharge.

mRS score	DAPT	Tirofiban	Total
0–2	11	23	34
3–5	3	14	17
6	1	3	4
Total	15	40	55

Abbreviation: DAPT, dual antiplatelet therapy.

**TABLE 6:** All patients' modified Rankin Scale (mRS) scores at the time of discharge.

mRS score	DAPT	Tirofiban	Total
0–2	52	281	333
3–5	10	58	68
6	1	15	16
Total	63	354	417

Abbreviation: DAPT, dual antiplatelet therapy.

**FIGURE 4:**
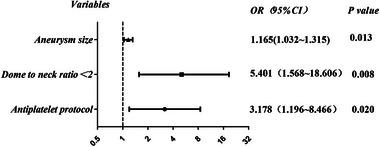
Multivariate logistic regression for risk factors of perioperative thromboembolic events (TEs) and hemorrhagic events (HEs). Multivariate logistic regression shows a forest plot of factors related to perioperative TEs and HEs, including perioperative antiplatelet protocols, dome‐to‐neck ratio < 2, and aneurysm size.

Logistic regression analysis was conducted to confirm the findings of this study. The results of our univariate analysis demonstrated that factors such as perioperative antiplatelet therapy, operative time, and dome‐to‐neck ratio < 2 were associated with perioperative TEs (*p* < .1), whereas age, sex, medical history, aneurysm location, and smoking and alcohol consumption were not (*p* > .1) (Table 7). Multifactorial logistic regression analysis was conducted to determine the factors independently associated with perioperative TEs following the initial univariate analysis. The results revealed that the use of an antiplatelet protocol (OR: 3.178, 95% CI: 1.196 to 8.466; *p *= .020 < .05) and dome‐to‐neck ratio < 2 (OR: 5.401, 95% CI: 1.568 to 18.606; *p* = .008 < .05) were significantly associated with perioperative TEs (Table 8 and Figure 4). Univariate analysis for perioperative HEs also found that aneurysm size and perioperative antiplatelet protocol were associated with perioperative HEs (*p* < .1), whereas preoperative platelet count, previous medical history, smoking and alcohol consumption, and dome‐to‐neck ratio < 2 were not significantly associated with perioperative HEs (*p* > .1) (Table 9). Multifactorial logistic regression analysis suggested that aneurysm size (OR: 1.165, 95% CI: 1.032 to 1.315; *p* = .013 < .05) was the sole factor independently associated with perioperative HEs (Table 8 and Figure 4), whereas antiplatelet protocols did not show a significant association.

**TABLE 7:** Univariable analyses of perioperative thromboembolic events (TEs).

Variables	*p*‐Value	OR (95% CI)
Age > 75	.914	0.921 (0.206–4.119)
Sex(male/female)	.544	0.544 (0.260–2.031)
Current smoking	.813	1.144 (0.375–3.491)
Current drinking	.294	1.946 (0.561–6.750)
Hypertension	.707	1.184 (0.492–2.852)
Diabetes	.633	1.436 (0.325–6.346)
Atherosclerosis	.936	0.964 (0.401–2.322)
WFNS grade	.932	1.067 (0.240–4.752)
WBC	.785	0.998 (0.179–2.547)
Aneurysm size	.445	1.067 (0.903–1.262)
Preoperative platelet count	.650	0.998 (0.991–1.006)
Antiplatelet protocols	.022	3.036 (1.174–7.852)
Aneurysm location	.676	0.728 (0.165–3.222)
Dome‐to‐neck ratio < 2	.006	5.246 (1.592–17.289)
Procedure time>4.1 h	.088	0.400 (0.140–1.145)
Number of treated aneurysms (1/2/3)	.562	0.675 (0.179–2.547)

Abbreviations: CI, confidence interval; OR, odd ratio; WFNS, World Federation of Neurological Society; WBC, white blood cell.

**TABLE 8:** Multivariable analyses of perioperative hemorrhagic events (HEs) and thromboembolic events (TEs).

Variables	Multivariable (TEs)		Multivariable (HEs)	
	*p*‐Value	OR (95%CI)	*p*‐Value	OR (95% CI)
Procedure time>4.1 h	.102	0.402 (0.135–1.197)		
Dome‐to‐neck ratio < 2	.008	5.401 (1.568–18.606)		
Antiplatelet protocol	.020	3.178 (1.196–8.466)	.080	2.053 (0.918–4.592)
Aneurysm size			.013	1.165 (1.032–1.315)

Abbreviations: CI, confidence interval; OR, odd ratio.

**TABLE 9:** Univariable analyses of perioperative hemorrhagic events (HEs).

	Univariable	
Variables	*p*‐Value	OR (95% CI)
Age > 75	.107	0.459 (0.178–1.185)
Sex (male/female)	.706	0.864 (0.405–1.844)
Current smoking	.733	1.161 (0.492–2.740)
Current drinking	.452	1.389 (0.590–3.266)
Hypertension	.690	0.870 (0.438–1.728)
Diabetes	.106	0.499 (0.215–1.158)
Atherosclerosis	.558	0.817 (0.416–1.606)
WFNS grade	.876	0.917 (0.308–2.728)
WBC	.556	0.974 (0.892–1.064)
Aneurysm size	.006	1.184 (1.049–1.336)
Preoperative platelet count	.804	0.999 (0.994–1.005)
Antiplatelet strategies	.038	2.285 (1.046–4.992)
Aneurysm location	.472	1.405 (0.556–3.551)
Dome‐to‐neck ratio < 2	.104	2.588 (0.823–8.142)
Procedure time>4.1 h	.853	1.108 (0.375–3.274)
Number of treated aneurysms (1/2/3)	.507	1.282 (0.615–2.672)

Abbreviations: CI, confidence interval; OR, odd ratio; WBC, white blood cell; WFNS, World Federation of Neurological Society.

## 4 DISCUSSION

In this retrospective analysis, we examined the impact of the use of antiplatelet protocols on the incidence of perioperative TEs and HEs and their prognostic implications in patients undergoing stent‐assisted embolization for ruptured intracranial aneurysms. The study revealed differences in the incidence of perioperative TEs and HEs based on the use of different antiplatelet protocols. The arteriovenous administration of tirofiban was effective in preventing perioperative TEs and did not increase the incidence of HEs. We conducted logistic regression analysis for perioperative TEs and HEs to support our findings and discovered that the dome‐to‐neck ratio < 2 was a protective factor for perioperative TEs, whereas aneurysm size was associated with perioperative HEs.

In this study, we analyzed the effect of antiplatelet protocol changes on TEs and HEs in the perioperative period for stent‐assisted embolization of ruptured aneurysms, including coagulation indicators and venous platelet counts in the first 24 h postoperatively and mRS scores at the time of discharge. Among 417 patients, the overall rate of perioperative TEs was approximately 6.4%; the rate of perioperative TEs was approximately 12.3% in the DAPT group and 5.3% in the tirofiban group. The disparity in the occurrence of perioperative TEs between the two groups suggests that tirofiban, administered through both arterial and venous routes, is effective in preventing perioperative thrombosis. Perioperative HEs occurred in 10 (15.87%) patients in the DAPT group and 27 (7.63%) patients in the tirofiban group; although the incidence of perioperative HEs was lower in the tirofiban group than in the DAPT group, multifactorial logistic regression revealed that the choice of the antiplatelet protocol was not a significant factor. Before the application of new platelet surface GP IIb/IIIa receptor antagonists such as tirofiban and abciximab in the endovascular therapy of intracranial aneurysms, a 3–5 day preoperative oral dual antiplatelet drug was often used to prevent perioperative TEs in patients with unruptured intracranial aneurysms. Present research supports the safety and efficacy of a loading dose of DAPT in preventing perioperative thrombotic events during endovascular treatment of ruptured intracranial aneurysms with stent assistance(Qoorchi Moheb Seraj et al., 2023). While the primary method for preventing perioperative TEs in endovascular therapy for patients with acutely ruptured aneurysms is preoperative oral DAPT, the rate of perioperative TEs is still high, ranging from approximately 8.33% to 13.21% (Wang et al., 2020; Zi‐Liang et al., 2017). Tirofiban was initially used as antiplatelet therapy for acute ischemic stroke, which resulted in a better prognosis (Investigators et al., 2022; Zi et al., 2023). Furthermore, in accordance with the recent updates in guidelines about antiplatelet and antithrombotic agents in neurointervention, there is a consensus recommendation for the utilization of tirofiban in the context of stent‐assisted embolization for the prophylaxis of perioperative ischemic events in ruptured aneurysms(Schirmer et al., 2023). Thereafter, a research team proposed a perioperative antiplatelet protocol for the prevention of perioperative TEs with tirofiban by a 3‐min intravenous infusion at 8 mg/kg and a subsequent 24‐h intravenous infusion of 0.1 ug/kg/min. They found that the incidence of perioperative TEs was 6.63%, a significant reduction with respect to the use of only a perioperative oral loading dose of tirofiban. Another meta‐analysis indicates comparable efficacy of GP IIb/IIIa inhibitors to DAPT in preventing perioperative TEs during endovascular treatment of intracranial aneurysms (Bilgin et al., 2022). However, GP IIb/IIIa inhibitors show a lower incidence of perioperative HEs compared to the DAPT group. Subgroup analysis within the GP IIb/IIIa inhibitor group reveals consistent rates of TEs and HEs regardless of aneurysm rupture. This study confirms the safety of GP IIb/IIIa inhibitors, like tirofiban, in endovascular embolization for intracranial aneurysms, with effectiveness similar to the DAPT group. The limited inclusion of relevant literature may contribute to the observed lack of significant differences. In addition, several studies have attempted to use arterial microcatheters to apply tirofiban to treat intraoperative acute thrombosis and prevent thrombus formation (Shen et al., 2022; Yoon et al., 2018), achieving better clinical efficacy. Therefore, our team implemented a perioperative antiplatelet protocol to prevent perioperative TEs with simultaneous intravenous pumping at a dose of 0.1 μg/kg/min for 24–48 h after pushing 5–20 mL of tirofiban via a guiding catheter after stent semi‐release. The findings indicated a decrease in perioperative TEs relative to the use of oral preoperative loading doses of dual antiplatelets. Additionally, the rate of HEs for the tirofiban group was lower than that of the DAPT group and similar to the effect of intravenous tirofiban in preventing TEs (Kim et al., 2016; Zi‐Liang et al., 2017). However, a multicenter retrospective study suggested that the prevention of perioperative TEs using intravenous tirofiban was less effective than when using a preoperative oral loading dose of DAPT. This could be due to the small sample size, with only 15 cases in the tirofiban group, making the study somewhat subject to error (Li et al., 2022). We further analyzed the postoperative 24‐h coagulation indexes and venous blood platelet counts. The data showed that the tirofiban group had better coagulation‐related indexes than the preoperative DAPT loading dose group, while no significant difference was seen in venous blood platelet counts. The correlation between coagulation indexes and patients' perioperative HEs suggested that perioperative HEs were lower with the combined use of arterial and venous tirofiban than with preoperative oral loading of the dual antiplatelet agents, while there was no significant effect on platelet counts. The comparison of mRS scores at discharge for patients in both groups did not show any significant differences, suggesting that the use of combined arteriovenous tirofiban did not have a significant impact on the near‐term prognosis of patients regarding preventing perioperative TEs. Regarding long‐term prognosis, a study showed that in stent‐assisted embolization for ruptured aneurysms, there was no significant difference in mRS scores at the 3‐month follow‐up between the DAPT group and the tirofiban group (Zi‐Liang et al., 2017). Additionally, when using intravenous tirofiban alone, the mRS scores at the time of discharge were not significantly different from those of the DAPT group (Zi‐Liang et al., 2017). However, the current study did not examine the long‐term prognosis of patients in either group, including regarding the impact of thromboprophylaxis with tirofiban. This may be a shortcoming of the current study.

Ultra‐early microthrombosis, occurring just 2 h after subarachnoid hemorrhage (SAH) onset, has been identified by magnetic resonance and pathological examination in a mouse model of SAH and has been supported by human pathological histological studies (Suzuki et al., 1990; Wang et al., 2021). The presence of microthrombosis is a significant factor in the development of early brain injury and delayed cerebral injury and may occur due to various factors (Zhou et al., 2022). Within 1 min after SAH onset, intracranial pressure (ICP) may rapidly increase to the mean arterial blood pressure, followed by a slow but still above‐normal decrease (Chen et al., 2022). The remarkable ICP increase results in a temporary stagnation of cerebral circulation or a severe reduction in cerebral blood flow through both arteries and veins. Hypoperfusion leads to systemic ischemia, causing generalized hypoxia, brain cell damage, and dysfunction. Complete ischemia disrupts the cerebral blood barrier by destroying endothelial cells, activating the coagulation cascade, and forming thrombotic clotting factors. All these factors contribute to the triad of stagnant blood flow, endothelial damage, and a hypercoagulable state, resulting in acute microthrombosis (Friedrich et al., 2012). Tirofiban is a rapid‐acting nonpeptide antagonist of platelet surface GP IIb/IIIa that facilitates plateau function recovery after the halt of antiplatelet drugs. This drug works according to three thrombolytic mechanisms, including preventing platelet aggregation, inhibiting the release of thrombolytic inhibitors from platelets, and destabilizing thrombi structurally. Tirofiban can stimulate the migration and proliferation of endothelial cells, thus repairing the vascular endothelium. This makes tirofiban more effective in preventing microthrombosis after subarachnoid hemorrhage. Studies have investigated the use of tirofiban after the stent‐assisted embolization of intracranial ruptured aneurysms to prevent delayed cerebral ischemia (Zanaty et al., 2021). Although the studies did not investigate early microthrombosis directly, the results suggest that reducing platelet activation may decrease microthrombosis. Furthermore, early microthrombosis is linked with stent‐assisted embolization for perioperative TEs. Thus, inhibiting microthrombosis may prevent TEs with the combined arterial and venous use of tirofiban during early stent‐assisted embolization of ruptured aneurysms for perioperative TEs.

To our knowledge, the use of combined arteriovenous tirofiban, an antiplatelet protocol, has been reported for the first time in the prevention of perioperative TEs during the stent‐assisted embolization of ruptured intracranial aneurysms. The results of this study indicate that the incidence of perioperative ischemic events was lower in the tirofiban group than in the preoperative loading DAPT group. However, the optimal dosing regimen for push and maintenance infusion of tirofiban remains unknown and requires further study.

In the treatment of intracranial aneurysms, wide‐necked aneurysms are typically defined by a dome‐to‐neck ratio < 2. It was concluded in this study that a dome‐to‐neck ratio < 2 provided protection against perioperative ischemic events during the stent‐assisted embolization of ruptured intracranial aneurysms. Additionally, patients with wide‐necked aneurysms had a lower perioperative ischemic event rate during stent‐assisted embolization, indicating the suitability of these structures for the procedure. Furthermore, it has been suggested that wide‐necked aneurysms are more amenable to stent‐assisted embolization. Several studies have suggested that the dome‐to‐neck ratio has an impact on perioperative TEs during stent‐assisted embolization of ruptured intracranial aneurysms (Li et al., 2022). Additionally, Liu et al. reported that an increase in the dome‐to‐neck ratio is associated with a higher likelihood of perioperative thromboembolic events during stent‐assisted embolization of ruptured intracranial aneurysms, although the association was not significant (Liu et al., 2016). However, no significant correlation was observed between the neck size of the aneurysm, aneurysm size, and perioperative TEs, consistent with the findings from other studies (Kim et al., 2016; Zi‐Liang et al., 2017). This research also found that aneurysm size was a risk factor for perioperative hemorrhage. The definition of perioperative HEs encompasses an array of hemorrhagic complications, which include perioperative intracranial hemorrhage, new postoperative gastrointestinal bleeding, hemoptysis, and other bleeding events. Other studies have demonstrated that aneurysm size is a high‐risk factor for perioperative bleeding events in stent‐assisted embolization of ruptured intracranial aneurysms (Koiso et al., 2023), and aneurysm size > 10 mm is more likely to be associated with perioperative intracranial hemorrhage, which may be because longer aneurysms are more likely to be associated with intraprocedural ruptured aneurysm bleeding.

There are also limitations in this study. First, the number of patients in the DAPT group was low, and this study was conducted in a single center. Moreover, to facilitate precise adjustment of the antiplatelet protocol, some patients did not undergo thromboelastography or antiplatelet drug sensitivity testing due to initial understanding limitations (Scott et al., 2013; Shuldiner et al., 2009). Platelet hyperreactivity is strongly linked with clinical ischemic events, while platelet hyporeactivity is linked with bleeding complications, as noted in previous studies (Flechtenmacher et al., 2015; Nishi et al., 2016). In the current study, we only counted cases of symptomatic perioperative thrombosis, without accounting for the possibility of asymptomatic thrombosis. Furthermore, the incidence of perioperative thromboembolisms may be higher because routine magnetic resonance diffusion‐weighted imaging was not conducted postoperatively. Additionally, differences in stent type and operator could have had an impact on the incidence of perioperative ischemic events.

## 5 CONCLUSION

In this single‐center retrospective study of perioperative complications in patients undergoing stent‐assisted embolization for ruptured intracranial aneurysms, the results showed that perioperative antiplatelet therapy with combined arterial and venous tirofiban was effective in preventing perioperative TEs and that there was no increased risk of perioperative HEs. This study also found that the dome‐to‐neck ratio < 2 was linked to perioperative TEs and aneurysm size was linked to perioperative HEs.

## AUTHOR CONTRIBUTIONS

**Kaishan Wang**: Methodology; investigation; data curation; writing—original draft. **Yujie Chen**: Conceptualization; methodology; writing—review and editing; supervision; funding acquisition. **Yao Xu**: Investigation; data curation. **Chen Yang**: Investigation; data curation. **Zhaopan Lai**: Investigation; data curation. **Binbin Tan**: Investigation; resources; supervision. **Gang Zhu**: Resources; supervision; funding acquisition. **Hongping Miao**: Conceptualization; methodology; writing—review and editing; supervision; funding acquisition. All authors read and approved the current version of the manuscript.

## CONFLICT OF INTEREST STATEMENT

The authors declare that they have no competing interests.

### PEER REVIEW

The peer review history for this article is available at https://publons.com/publon/10.1002/brb3.3439.

## Data Availability

The data that support the findings of this study are available on request from the corresponding author. The data are not publicly available due to privacy or ethical restrictions.
